# The Impact of COVID-19 on Preterm Birth Among Pregnant Women in Al-Qassim, Saudi Arabia

**DOI:** 10.7759/cureus.40682

**Published:** 2023-06-20

**Authors:** Lama S Alhumaidan, Nadiah Alhabardi, Sarah S Aldharman, Athar A Alfuhaid, May A Alrasheed, Rana S Almotairi, Joud A Alhassun, Ghaida a Alrohait, Reem F Almutairi, Farah S Alsuwailem, Aeshah M Alharbi, Lana R Alrashidi

**Affiliations:** 1 College of Medicine, Unaizah College of Medicine and Medical Sciences, Qassim University, Unaizah, SAU; 2 Department of Obstetrics and Gynecology, Unaizah College of Medicine and Medical Sciences, Qassim University, Unaizah, SAU; 3 College of Medicine, King Saud Bin Abdulaziz University for Health Sciences, Riyadh, SAU; 4 Department of Obstetrics and Gynecology, Maternity and Children Hospital, Burydah, SAU; 5 Department of Obstetrics and Gynecology, Qassim Health Cluster, King Saud Hospital, Qassim, SAU

**Keywords:** coronavirus in pregnancy, viral infections, prenatal covid-19 infection, preterm delivery, saudi arabia, covid-19

## Abstract

Background: Pregnant women are regarded as a unique group due to the distinct immunological condition that pregnancy produces, which makes pregnant women more susceptible to respiratory infections like coronavirus disease 2019 (COVID-19) and its consequences. During pregnancy, many viral infections have been recognized to increase the risk of adverse obstetrical outcomes such as preterm delivery. The purpose of this study was to investigate the effects of COVID-19 infection on preterm birth in pregnant women in the Al-Qassim region of Saudi Arabia.

Methods: This retrospective cohort study was conducted in Saudi Arabia between December 2019 to October 2021. The target subjects were pregnant women with live singleton gestations who underwent severe acute respiratory syndrome coronavirus 2 (SARS-CoV-2) polymerase chain reaction (PCR) testing for COVID-19 infection during their delivery hospitalization. Data gathered included patient demographic information, clinical characteristics, and pregnancy outcomes. Data were analyzed using R version 4.1.1 (R Core Team (2021); R Foundation for Statistical Computing, Vienna, Austria).

Results: A total of 381 pregnant women were included. The median maternal age of women was 31.0 years (IQR: 27.0, 35.0) and the median BMI value was 30.5 kg/m^2^ (IQR: 26.9, 34.8). The most common comorbidities were diabetes (7.1%) and asthma (4.5%). A known history of preterm birth was prevalent among 2.9%. Of the participants, 13.6% had a prenatal COVID-19 infection, of whom 57.7% had their infections resolved. The prevalence of positive PCR testing was 13.6%. Preterm birth occurred in 46 women (12.1%, 95%CI 9.1-15.9). Preterm birth was significantly associated with having a maternal age of ≥35 years, having high frequencies of parity, and having a past history of preterm birth, as well as having a history of hypertension and diabetes. Preterm birth was not significantly associated with having a confirmed COVID-19 infection at delivery.

Conclusion: It was shown that preterm birth is evident among women with COVID-19 infection. Preterm birth is significantly associated with old age, multiparity, and a history of preterm delivery. Preterm birth is not significantly associated with having a confirmed COVID-19 infection at delivery. More research regarding infection-related adverse effects is advised and should be highlighted.

## Introduction

In late 2019, a novel coronavirus emerged, spreading quickly from its place of origin in China across the globe. The Centers for Disease Control and Prevention (CDC) recommended terminology for the virus is severe acute respiratory syndrome coronavirus 2 (SARS-CoV-2), and the illness caused by this virus is called coronavirus disease 2019 (COVID-19). COVID-19 has a wide range of clinical symptoms, which are divided into five categories: non-symptomatic (1.2%), mild to moderate (80%), severe (13%), critical (4%), and fatal (2%). COVID-19 is more common among reproductive-age young adults than in older adults [1]. However, the existing information on COVID-19's impact on pregnant women is still insufficient [2]. According to the Saudi Ministry of Health, there is limited data available on COVID-19 during pregnancy that suggest an increased risk of miscarriage or preterm birth if a pregnant woman is infected with COVID-19 [3].

Pregnant women are considered a unique category due to the specific immunological status that pregnancy causes, making pregnant women more vulnerable to respiratory infections such as COVID-19 and their implications [4]. Furthermore, during pregnancy, many viral infections have been recognized to raise the risk of adverse obstetrical outcomes, including preterm delivery. Preterm birth is a danger reported in a number of studies at varying rates. According to the first data on the obstetrical outcomes of pregnant women with COVID-19, preterm births significantly increased [1]. A recent prospective cohort study shows Infected pregnant women had somewhat high rates of preterm birth, preeclampsia, and cesarian section (CS) [5]. In addition, pregnant women with COVID-19 were more likely to need intensive care than non-pregnant women of reproductive age [6]. According to a report issued by the CDC, women who have COVID-19 during pregnancy are more inclined to require ICU care while hospitalized [7].

Moreover, there are some factors that we need to consider that are related to the severity of symptoms of COVID-19 in pregnancy, such as high body mass index (BMI), comorbidities, and age of the mother; all of these factors are linked to serious complications and might necessitate admission to an ICU, invasive ventilation (IV), and maternal death [6]. In determining the risk of preterm birth in symptomatic versus asymptomatic pregnant women, a recent systematic review and meta-analysis study discovered substantial differences between the two groups, with symptomatic pregnant women having more preterm births and a higher likelihood of maternal ICU admission [8]. Moreover, according to a study conducted in Latifa Hospital in Dubai, pregnant women have a severe course of illness compared to non-pregnant women with COVID-19 infections with a statistically significant worse chest radiograph score and ICU admission. They concluded that pregnancy with COVID-19 could be categorized as a high-risk pregnancy [9]. 

Although many complications could be linked to COVID-19 infection, preterm birth is one of the most common. Between March and November 2020, a retrospective cohort and multicenter research of COVID-19-infected pregnant women was done at three designated hospitals in Riyadh, Saudi Arabia. During the study period, 288 pregnant women with confirmed COVID-19 infection were identified with preterm birth (15.5%) as the most common adverse pregnancy outcome [2]. Severe maternal morbidity as a result of COVID-19 and perinatal deaths were reported in the literature [10]. Another systematic review and meta-analysis of the outcome of coronavirus spectrum infection during pregnancy found that the infected mothers who also had pneumonia had preterm birth as the most common adverse outcome [11]. A systematic review evaluating the pregnancy outcomes, including 10 Chinese studies, two from the US, and one Italian study, found a preterm birth rate of 20.1% [12]. Another prospective cohort study in the UK found that of 427 pregnant women hospitalized with confirmed COVID-19 infection, 27 % of them had a preterm birth [13]. All of these findings suggest an increased risk of complications for pregnant women related to COVID-19 infection. Patients with comorbidities have a higher risk of a worse prognosis, including pregnant women. Therefore, it is important to carefully construct a long-term follow-up strategy for the children of COVID-19-affected expectant mothers. As mentioned before, many studies have suggested that COVID-19 was associated with maternal morbidity and has been linked to preterm birth. However, we found during our literature review that there are insufficient studies about COVID-19's impact on pregnant women in Saudi Arabia.

COVID-19 has been associated with poor prognosis and unfavorable maternal outcomes. One of these primary outcomes is preterm labor [13]. Few studies were done in Saudi Arabia to find out whether there is an association between COVID-19 and preterm labor. More focus is needed concerning this topic to determine the incidence of preterm labor and optimize suitable healthcare. The purpose of this study was to investigate the effects of COVID-19 infection on preterm labor in COVID-19-positive pregnant women in the Al-Qassim region of Saudi Arabia. This study will serve to implement preventative measures to avoid life-threatening situations that may occur to the mother. Even though there are studies done in this field of research, the clinical correlation between preterm labor and COVID-19 infection is yet to be confirmed in the Al-Qassim region, and the study will assist physicians in providing necessary healthcare to such patients.

## Materials and methods

This retrospective cohort study was conducted between December 2019 to October 2021 in three hospitals of the Al-Qassim region of Saudi Arabia, namely: Maternity and Children Hospital in Buraydah, King Saud Hospital in Unaiza, and Dr. Sulaiman Al Habib Hospital in Buraydah. The inclusion criteria were pregnant women with live singleton gestations who underwent SARS-CoV-2 polymerase chain reaction (PCR) testing for COVID-19 infection during their delivery hospitalization. Any pregnancy with multiple gestations or stillbirth or who did not undergo SARS-CoV-2 PCR testing during their delivery hospitalization was excluded. 

Sample size and sampling technique 

OpenEpi® version 3.01 software was employed to calculate the sample size. The representative sample size required was 385 with a margin error determined as 5%, a confidence level determined as 95%, and the population proportion determined as 50%. A purposive sampling technique has been employed.

Data collection

All patients to whom our inclusion and exclusion criteria were applied had their charts reviewed and data collected by the co-investigators of the research through a data collection sheet on Excel (Microsoft Corporation, Redmond, Washington, United States). Data were collected using patients' medical records. Patient demographics, clinical features, and pregnancy outcomes such as gestational age at birth were gathered in the data collection. The classification of preterm births as spontaneous or medically indicated was done by reviewing patient data. Additionally, records were examined to categorize the level of disease for participants who had a positive COVID-19 infection. 

In the data collecting sheet, the following variables were listed: age of the mother, the number of children, previous preterm births, BMI, the status of marriage, hypertension, pulmonary diseases such as asthma or persistent hypertension, diabetes (pre- or gestational), gestational hypertension, preeclampsia, HELLP (hemolysis, elevated liver enzymes, and low platelets) syndrome, eclampsia, and month of delivery (In weeks). Additionally, we gathered the infection status for COVID-19: infection at delivery (based on positive SARS-CoV-2 PCR testing during delivery hospitalization) or resolved prenatal infection (based on positive IgG antibody testing or prior positive PCR testing, and negative SARS-CoV-2 PCR testing). Also, we collected the severity of COVID-19 infection (asymptomatic, mild, moderate, severe), outcome (preterm birth/ term), and preterm birth (spontaneous/medically indicated)

All information was kept confidential. Informed permission was given by all participants prior to filling out the questionnaire, and participation in the study was voluntary. Ethical approval was obtained from the Regional Bioethics Committee in Al-Qassim Health Authority (approval number: 607-43-6008).

Statistical analysis

Data were analyzed using R version 4.1.1 (R Core Team (2021); R Foundation for Statistical Computing, Vienna, Austria). Frequencies and percentages were used to express categorical data, and numerical variables were presented as median and interquartile range (IQR). The prevalence rates of COVID-19 infection and preterm birth were analyzed using a one-sample proportion test with continuity correction, and the outcome was expressed as prevalence and the respective 95% confidence interval (95% CI). The factors associated with preterm birth were analyzed using a Wilcoxon rank sum test for numerical variables and Fisher's exact test for categorical data. A multivariate logistic regression model was constructed to assess the possible risk factors for preterm birth. We used the significantly associated variables from the association analysis as independent variables and the preterm birth variable (no versus yes) as a dependent variable. The outcomes were presented using odds ratio (OR) and 95%CI. A p-value of < 0.05 indicated statistical significance.

## Results

Demographic and clinical characteristics

Initially, we collected data of 385 subjects in the current study. However, we excluded the records of four women who had an unknown status of COVID-19 infection at delivery. Therefore, data of 381 women were ultimately analyzed. The median maternal age of women was 31.0 years (IQR: 27.0, 35.0) and the median BMI value was 30.5 kg/m^2^ (IQR: 26.9, 34.8). More than half of the participants were obese (54.2%). All the women were married. The most common comorbidities were diabetes (7.1%) and asthma (4.5%). A known history of preterm birth was prevalent among 2.9%. Of note, 13.6% of the participants had a prenatal COVID-19 infection, of whom 57.7% had their infections resolved. Details of demographic and clinical characteristics are given in Table 1.

**Table 1:** Demographic and clinical characteristics (n=381) Missing data for weight (n=8), height (n=7), and BMI (n=10) IQR: interquartile range

Parameter	Category	n (%)
Maternal age (years), median (IQR)		31.0 (27.0, 35.0)
Maternal age ≥35 years	Yes	115 (30.2%)
Parity	0	71 (18.6%)
	1	103 (27.0%)
	2	82 (21.5%)
	≥3	125 (32.8%)
Weight (kg), median (IQR)		77.0 (67.0, 87.0)
Height (cm), median (IQR)		159.0 (155.0, 162.0)
BMI (kg/m^2^), median (IQR)		30.5 (26.9, 34.8)
BMI ≥30	Yes	201 (54.2%)
Comorbidities	Chronic hypertension	11 (2.9%)
	Diabetes	27 (7.1%)
	Asthma	17 (4.5%)
	Other chronic pulmonary disease	8 (2.1%)
	Hypertensive disorders of pregnancy	17 (4.5%)
History of preterm birth	No	310 (81.4%)
	Yes	11 (2.9%)
	Unknown	60 (15.7%)
Gestational age at delivery (weeks), median (IQR)		39.0 (38.0, 40.0)
Prenatal infection	Yes	52 (13.6%)
Resolved prenatal infection	No	22 (42.3%)
	Yes	30 (57.7%)

Characteristics of COVID-19 infections

**Figure 1:**
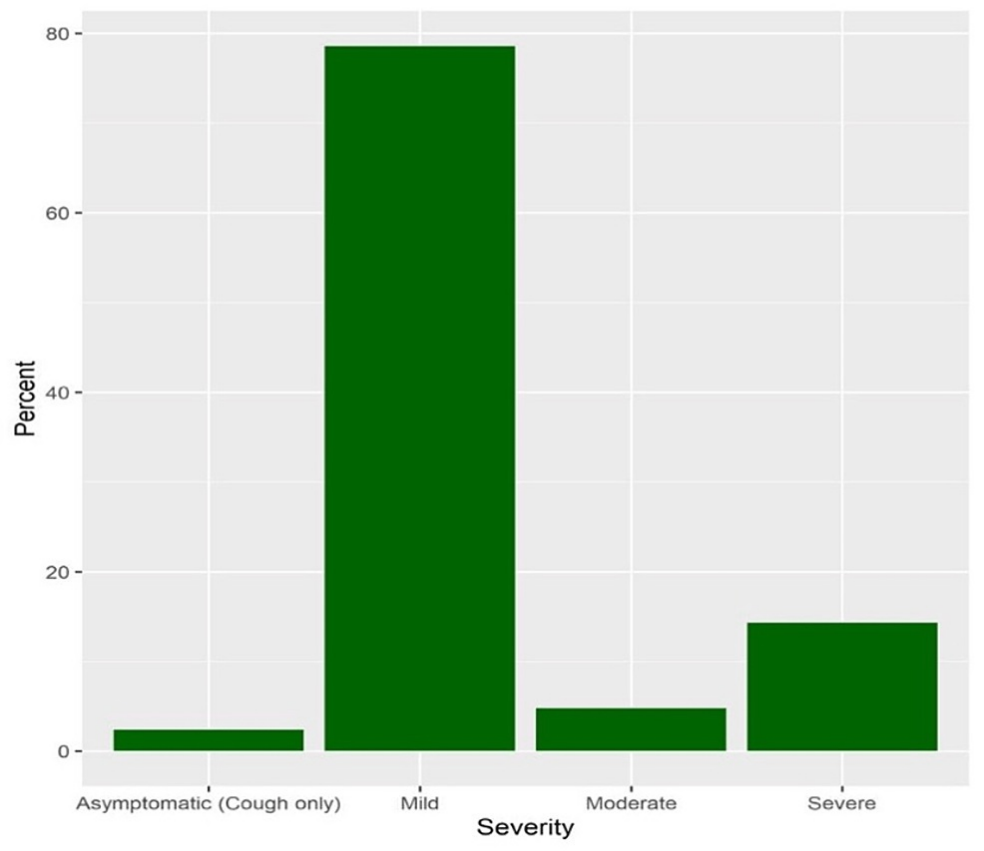
COVID-19 severity COVID-19: coronavirus disease 2019

A total of 52 women had positive PCR testing for COVID-19 infection, with a prevalence of 13.6% (95%CI 10.4-17.6). The majority of infections were mild (78.6%), while 4.8% and 14.3% had moderate and severe infections, respectively (Figure 1).

Characteristics of preterm birth

**Figure 2:**
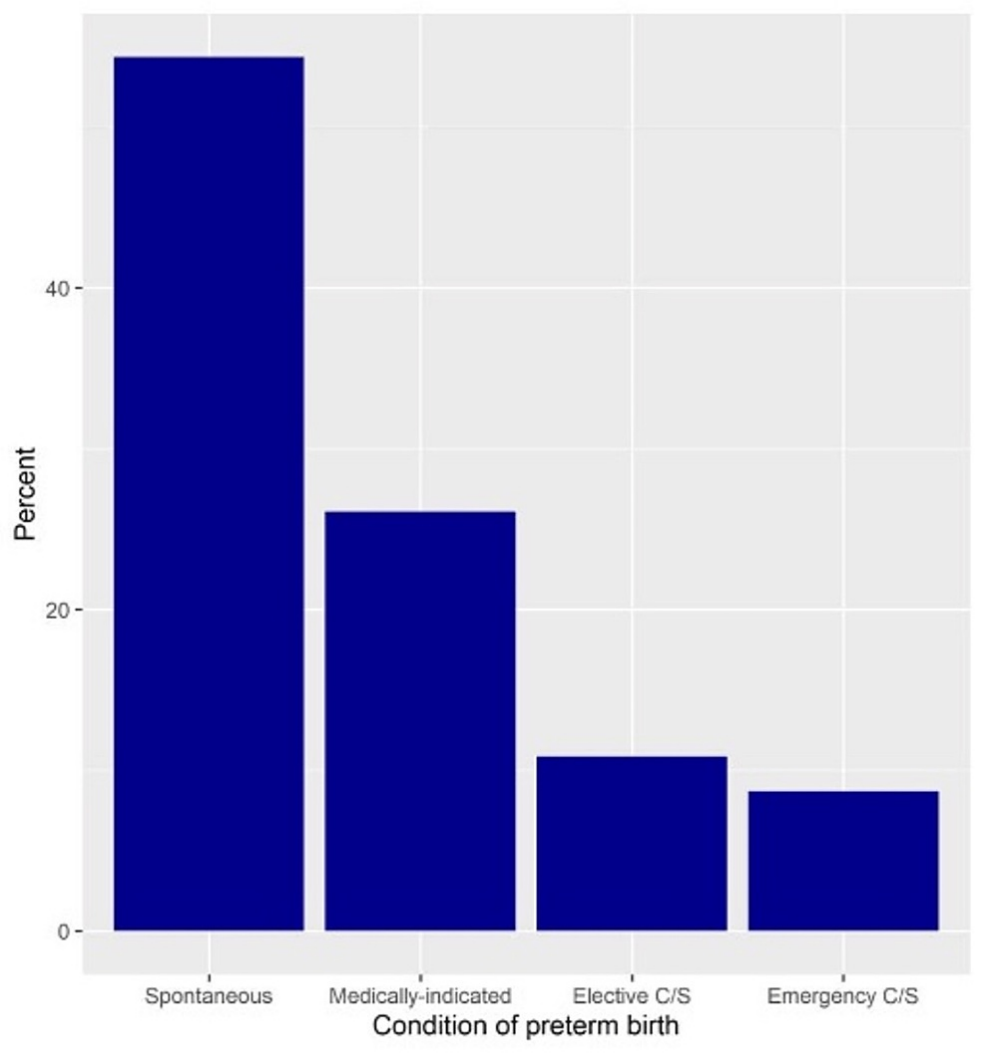
Conditions of preterm deliveries C/S: cesarian section

Preterm birth occurred in 46 women (12.1%, 95%CI 9.1-15.9). Among those with preterm birth, spontaneous birth occurred in 54.3%, medically-indicated birth in 26.1%, elective CS in 10.9%, and emergency CS in 8.7% (Figure 2).

Factors associated with preterm birth

Preterm birth was significantly associated with a maternal age of ≥35 y (45.7% vs 28.1% among those without preterm birth, p = 0.025), high frequencies of parity (41.3% had ≥3 parity vs 1.2% among those without preterm birth, p = 0.040), a past history of preterm birth (15.2% vs 1.2% among those without preterm birth, p < 0.001), and a history of hypertension (8.7% vs 2.1% among those without preterm birth, p = 0.033) and diabetes (15.2% vs 6.0% among those without preterm birth, p = 0.032). Preterm birth was also associated with lower gestational age at delivery (median = 35 weeks, IQR = 34, 36 vs median = 39 weeks, IQR = 38, 40 among those without preterm birth, p < 0.001). Importantly, preterm birth was not significantly associated with having a confirmed COVID-19 infection at delivery (8.7% vs 14.3% among those without preterm birth, p = 0.366, Table 2).

**Table 2:** Factors associated with preterm birth IQR: interquartile range

Parameter	Category	Preterm birth		
		No, N = 335	Yes, N = 46	p-value
Maternal Age (y)	Median (IQR)	31.0 (27.0, 35.0)	33.5 (29.0, 38.0)	0.011
Maternal age >=35 y	Yes	94 (28.1%)	21 (45.7%)	0.025
Parity	0	68 (20.3%)	3 (6.5%)	0.040
	1	93 (27.8%)	10 (21.7%)	
	2	68 (20.3%)	14 (30.4%)	
	≥3	106 (31.6%)	19 (41.3%)	
BMI	Median (IQR)	30.5 (27.0, 34.9)	29.9 (26.6, 33.7)	0.478
BMI >=30	Yes	179 (54.7%)	22 (50.0%)	0.629
Comorbidities	Chronic Hypertension	7 (2.1%)	4 (8.7%)	0.033
	Diabetes	20 (6.0%)	7 (15.2%)	0.032
	Asthma	16 (4.8%)	1 (2.2%)	0.706
	Other chronic pulmonary disease	7 (2.1%)	1 (2.2%)	>0.999
	Hypertensive disorders of pregnancy	13 (3.9%)	4 (8.7%)	0.136
History of preterm birth	No	284 (84.8%)	26 (56.5%)	<0.001
	Yes	4 (1.2%)	7 (15.2%)	
	Unknown	47 (14.0%)	13 (28.3%)	
Gestational age at delivery (weeks)	Median (IQR)	39.0 (38.0, 40.0)	35.0 (34.0, 36.0)	<0.001
Prenatal infection	Yes	48 (14.3%)	4 (8.7%)	0.366
Resolved prenatal infection	Resolved	27 (8.1%)	3 (6.5%)	0.648
	Not resolved	21 (6.3%)	1 (2.2%)	
COVID-19 Infection at delivery	Yes	48 (14.3%)	4 (8.7%)	0.366

Risk factors for preterm birth

**Figure 3:**
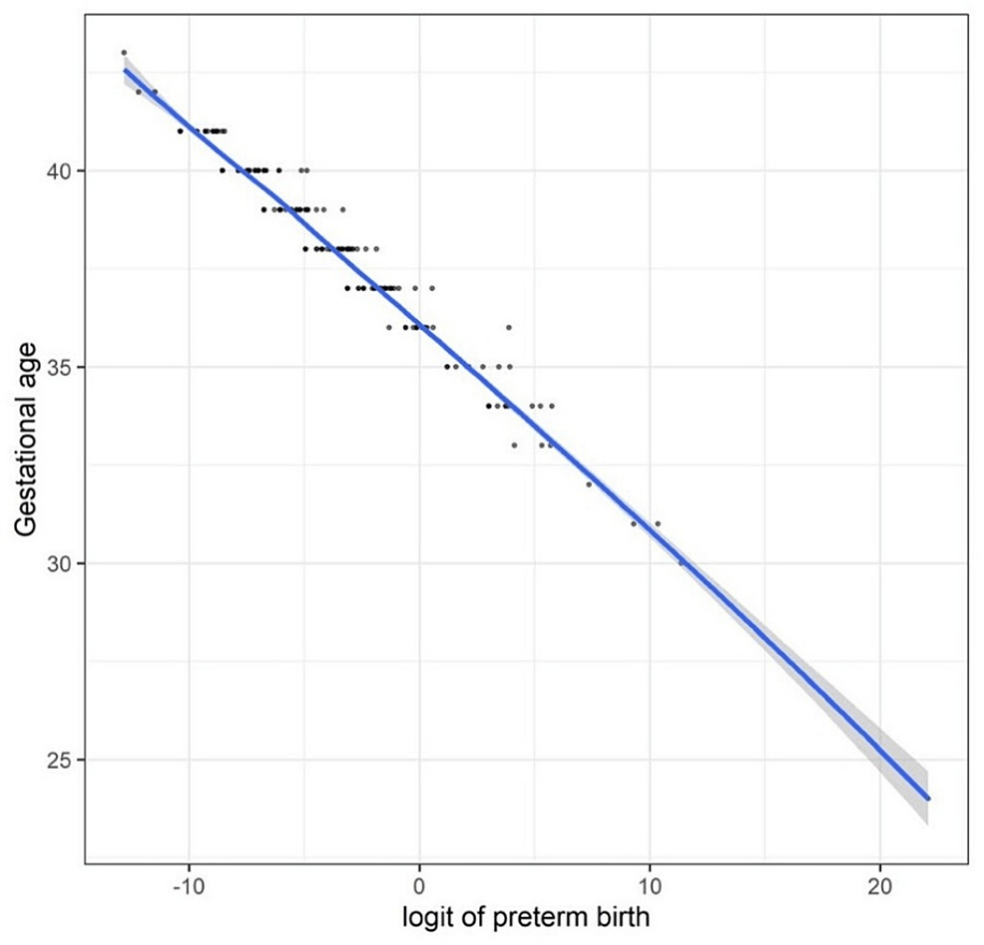
A scatter plot depicting the relationship between gestational age and the logit of preterm birth

The following were independent variables: maternal age, parity, chronic hypertension, diabetes, a past history of preterm birth, and gestation age at delivery. Model assumptions were satisfied since the risk of multicollinearity was not evident (a variance inflation factor was < 5 for all independent variables) and gestational age was linearly correlated with the logit values of the outcome variable (preterm birth) (Figure 3). Results of the regression analysis showed that the lower gestational age was the sole independent risk factor for preterm birth (OR = 0.16, 95%CI 0.09-0.26, p < 0.001) (Table 3).

**Table 3:** Results of the regression analysis for the risk factors of preterm birth

Parameter	Category	OR	95% CI	p-value
Maternal age ≥35 y	No	—	—	
	Yes	1.62	0.44, 6.01	0.464
Parity	0	—	—	
	1	4.17	0.47, 71.3	0.253
	2	2.01	0.19, 34.7	0.587
	>=3	2.02	0.23, 32.5	0.569
Chronic hypertension	No	—	—	
	Yes	2.84	0.17, 27.6	0.404
Diabetes	No	—	—	
	Yes	1.59	0.33, 6.83	0.546
History of preterm birth	No	—	—	
	Yes	9.46	0.84, 154	0.083
	Unknown	1.46	0.35, 5.65	0.591
Gestational age at delivery (weeks)	Numerical	0.16	0.09, 0.26	<0.001

## Discussion

Preterm birth (birth before 37 weeks of gestation) is a major reason for mortality and morbidity during the neonatal period, childhood, and adulthood, and the causes behind it are multi-factorial. We included data of 381 women. The most commonly reported comorbidities were diabetes (7.1%) and asthma (4.5%). A known history of preterm birth was prevalent among 2.9%. Of note, 13.6% of the participants had a prenatal COVID-19 infection, of whom 57.7% had their infections resolved.

Pregnant women are highly susceptible to respiratory infections due to the adaptive anatomical and physiological responses in the respiratory system that happen during pregnancy, and these viral infections may cause pregnancy complications [14]. This is compatible with Oltean et al.’s review where elevated rates of preterm birth in women with COVID-19 were reported in comparison to pregnant women without SARS-CoV-2 infection [15]. A multi-center study by Al-Matary et al. in Saudi Arabia identified a total of 288 pregnant women with confirmed COVID-19 infection. The most common adverse pregnancy outcome was premature birth (n = 31, 15.5%) [2]. In our study, preterm birth occurred in 46 women (12.1%).

Similarly, another systematic review study and meta-analysis of the outcome of coronavirus spectrum infection during pregnancy found that the infected mothers who also had pneumonia had a preterm birth as the most common adverse outcome [11]. Moreover, a systematic review evaluating the pregnancy outcomes, including 10 Chinese studies, two from the United States, and one from Italy found a preterm birth rate of 20.1% [12]. 

In addition to the direct impact of COVID-19 on pregnancy and its results, there is evidence that the pandemic and its impact on healthcare systems have had major consequences on pregnancy outcomes, including an increase in stillbirths and maternal death. These patterns might signify growing gaps and a worrisome reversal of recent advancements in mother and newborn health. Our results observed that preterm birth was significantly associated with having a maternal age of ≥35 years, multiparity, and having a past history of preterm (p-value > 0.05).

We observed that preterm birth was not significantly associated with having a confirmed COVID-19 infection at delivery (p-value <0.05). This is contrary to the findings of several previous studies that reported a direct association between preterm birth and COVID-19 infection [12]. In a previous study, the severity of COVID-19 symptoms in women was associated with more susceptibility to adverse outcomes such as premature birth [11]. While several studies have reported an increase in preterm birth among women infected with COVID-19, others reported a decline in the rates. For instance, reduced rates of preterm birth were reported in Denmark [16]. Preterm birth rates have been shown to decrease for a variety of causes, including less physical activity during pregnancy, lessened stress related to work-life balance, decreased exposure to infection, fewer medical interventions, and increased hygiene and rest. Increases in premature birth rates may be caused by stress brought on by fear about the epidemic, difficulties with jobs or finances, homestays, and restricted maternity care [17].

In addition, safety and precaution measures implemented when getting the infection, as well as the stigma and denial, would reduce the probability of getting medical care in a tertiary center, which would later lead to various complications and consequences either at delivery or postpartum.

We consider this study a valuable base for evidence as it is one of the first to be conducted in the Al-Qassim region of Saudi Arabia. Another strength is that it compared pregnancy outcomes between patients with and without infection from the same population and time period. However, a limitation of this study is that it was conducted in a selected area of Saudi Arabia, which may affect the generalization of the findings. Furthermore, some of these studies are vulnerable to selection bias by over-inclusion of symptomatic cases with more severe illness, as well as recall bias since data were collected from records. We recommend that serial and frequent studies on the issue should be conducted to generate more evidence and data regarding this topic. Also, evidence-based data regarding COVID-19 and its effect on pregnancy outcomes should be displayed and spread through all media platforms to females in the community. 

## Conclusions

It was found that preterm birth is evident among women with COVID-19. Preterm birth is significantly associated with having old age, multiparity, and having a history of preterm. Preterm birth is not significantly associated with having a confirmed COVID-19 infection at delivery. We recommend that future studies on the issue should be conducted to build more evidence and data regarding this topic.
